# Defining the pharmacokinetic/pharmacodynamic index of piperacillin/tazobactam within a hollow-fibre infection model to determine target attainment in intensive care patients

**DOI:** 10.1093/jacamr/dlae036

**Published:** 2024-03-12

**Authors:** Suzanne A M Wenker, Najla Alabdulkarim, John B Readman, Elise M A Slob, Giovanni Satta, Shanom Ali, Nishma Gadher, Rob Shulman, Joseph F Standing

**Affiliations:** Infection, Immunity and Inflammation Department, Great Ormond Street Institute of Child Health, University College London, London, UK; Department of Clinical Pharmacy and Toxicology, Leiden University Medical Center, Leiden University, Leiden, The Netherlands; Infection, Immunity and Inflammation Department, Great Ormond Street Institute of Child Health, University College London, London, UK; Department of Clinical Pharmacy, Princess Nourah bint Abdulrahman University, Riyadh, Saudi Arabia; Infection, Immunity and Inflammation Department, Great Ormond Street Institute of Child Health, University College London, London, UK; Department of Clinical Pharmacy and Toxicology, Leiden University Medical Center, Leiden University, Leiden, The Netherlands; Department of Clinical Pharmacy, Haaglanden Medical Center, The Hague, The Netherlands; Department of Infection, University College London Hospitals NHS Foundation Trust, London, UK; Environmental Research Laboratory, University College London Hospitals NHS Foundation Trust, London, UK; Pharmacy Department, CMORE, University College London Hospitals NHS Foundation Trust, London, UK; Pharmacy Department, CMORE, University College London Hospitals NHS Foundation Trust, London, UK; Infection, Immunity and Inflammation Department, Great Ormond Street Institute of Child Health, University College London, London, UK; Department of Pharmacy, Great Ormond Street Hospital for Children, London, UK

## Abstract

**Background:**

It is important to optimize dosing schemes of antibiotics to maximize the probability of therapeutic success. The recommended pharmacokinetic/pharmacodynamic (PK/PD) index for piperacillin/tazobactam therapy in clinical studies ranges widely (50%–100% *fT*_>1–4×MIC_). Dosing schemes failing to achieve PK/PD targets may lead to negative treatment outcomes.

**Objectives:**

The first aim of this study was to define the optimal PK/PD index of piperacillin/tazobactam with a hollow-fibre infection model (HFIM). The second aim was to predict whether these PK/PD targets are currently achieved in critically ill patients through PK/PD model simulation.

**Patients and methods:**

A dose-fractionation study comprising 21 HFIM experiments was performed against a range of Gram-negative bacterial pathogens, doses and infusion times. Clinical data and dose histories from a case series of nine patients with a known bacterial infection treated with piperacillin/tazobactam in the ICU were collected. The PK/PD index and predicted plasma concentrations and therefore target attainment of the patients were simulated using R version 4.2.1.

**Results:**

*fT*_>MIC_ was found to be the best-fitting PK/PD index for piperacillin/tazobactam. Bactericidal activity with 2 log_10_ cfu reduction was associated with 77% *fT*_>MIC_. Piperacillin/tazobactam therapy was defined as clinically ‘ineffective’ in ∼78% (7/9) patients. Around seventy-one percent (5/7) of these patients had a probability of >10% that 2  log_10_ cfu reduction was not attained.

**Conclusions:**

Our dose-fractionation study indicates an optimal PK/PD target in piperacillin/tazobactam therapies should be 77% *fT*_>MIC_ for 2 log_10_ kill. Doses to achieve this target should be considered when treating patients in ICU.

## Introduction

The increasing incidence of global antimicrobial resistance reported in bacterial human pathogens in combination with the lack of antimicrobial development success in recent decades has raised concerns about antimicrobial treatment options for the future. For critically ill patients admitted to the ICU, concerns have been raised about current antibiotic treatment failure. Dosing regimens of antibiotics are usually developed in healthy volunteers and ward patients; however, ICU patients may exhibit different pharmacokinetic (PK) characteristics.^1^

Dosing schemes of antibiotics are defined using the PK/pharmacodynamic (PK/PD) index. Currently, antibiotics are classified into three different PK/PD indices: (i) the ratio between the maximum drug concentration reached compared to the MIC (*fC*_max_/MIC); (ii) the ratio between the AUC_24_ compared with the MIC (*f*AUC/MIC); or (iii) the fraction of time that the antibiotic concentration exceeds the MIC (*fT*_>MIC_). The PK/PD index is usually determined with dose-fractionation studies.

Piperacillin/tazobactam is a penicillin/β-lactamase inhibitor combination prescribed frequently in the hospital setting and with a broad spectrum of activity for the treatment of a diverse range of infections. For piperacillin/tazobactam, a wide range of PK/PD targets in clinical studies have been described (50%–100% *fT*_>1–4×MIC_).^2–6^

Dose-fractionation experiments of piperacillin/tazobactam have been performed using a neutropenic murine thigh infection model,^7^ a one-compartment *in vitro* model,^8^ and a hollow-fibre infection model (HFIM).^9^ A limitation of some of these experiments was that only the *Escherichia coli* strains were assessed, which does not account for piperacillin/tazobactam therapies used for more causative agents in clinical practice.^7,8^ Further, in some experiments, piperacillin/tazobactam was not evaluated in its clinically employed 8:1 ratio,^10^ since piperacillin concentrations were fixed whilst tazobactam concentrations varied.^8,9^ Additionally, these experiments only evaluated 30 min infusions.^7–9^

The first aim of this study was to undertake a dose-fractionation study in an HFIM to define the PK/PD index of piperacillin/tazobactam against several clinically relevant Gram-negative bacteria. Upon determining the PK/PD index and targets, the second aim was to demonstrate whether PK/PD targets are likely to be attained in ICU patients and to assess the outcomes of current treatment of known infections.

## Materials and methods

A dose-fractionation study was performed using an HFIM. For details on the materials, HFIM setup and PK validation method, see the Supplementary Methods (available as Supplementary data at *JAC-AMR* Online).

### Method for dose-fractionation assays

The MICs were determined using the broth microdilution method.^11^ Seven growth control experiments and 14 dose-fractionation experiments were performed. The drug infusion was administered every 6 h over 24 h. Infusion times were 30 min or 3 h.

Before the start of the experiments, the bacterial titre as cfu/mL in the inoculum was estimated using a UV spectrophotometer to measure OD. During the experiments, the cfu/mL was measured by diluting samples 10-fold in PBS and plating them out on Mueller–Hinton (MH) agar plates.

The PK/PD index and targets were defined using the statistical software R version 4.2.1. Non-linear least squares were used to build models on the 24 h timepoint with model selection guided by Akaike information criteria (AIC) and R^2^.

### Target attainment

Clinical data for patients admitted to the ICU, University College London Hospitals from 27 July 2021 to 30 July 2022 were collected. Ethical approval was not deemed necessary as this was registered as an internal audit (a non-interventional retrospective audit of practice). One database had a collection of patients with known infections of either *E. coli*, *Klebsiella pneumoniae* or *Pseudomonas aeruginosa.* The other database consisted of patients who were administered piperacillin/tazobactam therapy in the ICU. The databases were combined to select patients who had a positive blood culture of one of the strains during piperacillin/tazobactam treatment in the ICU. The dosing scheme (q6h versus q8h), the change in serum creatinine during the dosing scheme, age, weight, sex and whether they were on haemofiltration were collected. To predict blood concentrations for each patient, primary PK parameters of a previously made PK/PD model by Lonsdale *et al.*^3^ were used. Since patients on haemofiltration were excluded from that study, the dialysis clearance parameter defined by the PK/PD model of ertapenem by Eyler *et al.*^12^ was added. With these primary parameters and the individual parameters of each patient, the piperacillin/tazobactam concentration was simulated 1000-fold in R version 4.2.1. Subsequently, it was predicted whether the PK/PD targets were reached. As a reference point to define the MICs per isolate, the epidemiological cut-off (ECOFF) value defined by EUCAST for piperacillin/tazobactam was used.^13^

The outcome of piperacillin/tazobactam treatment per patient was classified as ‘effective’, ‘ineffective’ or ‘indeterminant’. ‘Ineffective’ was defined as starting additional antibiotics with a broader or similar spectrum within 48 h after treatment or death due to infection within 10 days after the end of treatment. Treatment was defined as ‘indeterminate’ if the dosing scheme was too short to be able to have an effect or if the patient died of other causes during therapy. The outcome was defined as ‘effective’ in any other situation.

## Results

### Dose-fractionation study

**Figure 1.**
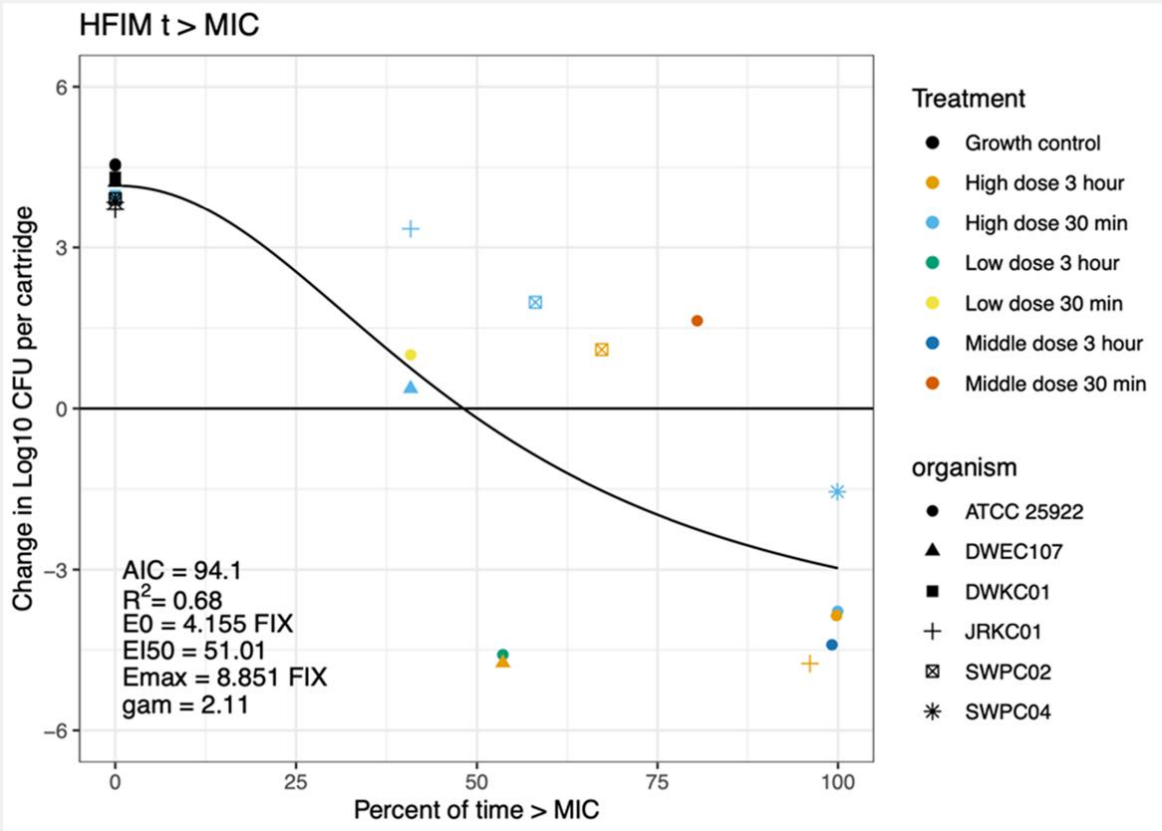
Model for PK/PD index *fT*_>MIC_ for piperacillin/tazobactam. The black line indicates the PK/PD index model *fT*_>MIC_. The dots represent the outcomes of each experiment. The closer the dots are to the black line, the better the model fits. The different colours of the dots denote the doses used, as defined in Materials and methods; shapes denote the organism used (*E. coli*: ATCC25922, DWEC107; *K. pneumoniae*: DWKC01, JRKC01; *P. aeruginosa*: SWPC02, SWPC04). The *x*-axis represents the percentage of time the piperacillin/tazobactam concentration was above the MIC; the *y*-axis represents the change in log_10_ cfu/mL over 24 h.

The MICs were 2, 32, 256, 32, 64 and 4 mg/L for ATCC 25922, DWEC107 (*E. coli*), DWKC01, JRKC01 (*K. pneumoniae*), SWP02 and SWPC04 (*P. aeruginosa*), respectively. For the results of the growth control and dose-fractionation experiment, see Figure S1. Figure 1 depicts the best-fitting PK/PD model. The AIC for *fT*_>MIC_ was 94.1 with an R^2^ value of 0.691, whereas the corresponding values for *fC*_max_/MIC and *f*AUC/MIC were 102/0.526 and 96.9/0.62, respectively. The PK/PD targets for *fT*_>MIC_ were as follows: bacteriostasis was associated with 48% *fT*_>MIC_, 1 log_10_ kill was associated with 60% *fT*_>MIC_ and 2 log_10_ kill with 77% *fT*_>MIC_.

### Target attainment

Nine patients were selected, with 4/9 treated with piperacillin/tazobactam 4.5 g q6h and 5/9 patients treated with 4.5 g q8h. The treatment was noted as ‘effective’ in one patient, ‘indeterminate’ in one patient and ‘ineffective’ in seven patients (Table 1). For five patients, all with ‘ineffective’ treatment outcome, there is a probability of 10% or more that the target for 2 log_10_ kill was not reached. There was no statistical difference between the q6h and q8h groups (unpaired *t*-test, *P* value = 0.9062).

**Table 1.** Patient characteristics, treatment outcomes and % probability of PK/PD target attainment per patient

Patient	Age	Weight (kg)	Sex	Serum creatinine range (μmol/L)	Renal replacement therapy	Treatment outcome	Organism	Dosing scheme (4.5 g piperacillin/tazobactam, 30 min infusion)	*fT*_>MIC_ (median)	% PTA bacteriostasis (48% *fT*_>MIC_)	% PTA 1 log_10_ kill (60% *fT*_>MIC_)	% PTA 2 log_10_ kill (77% *fT*_>MIC_)
1	84	50	male	73–154	no	ineffective	*E. coli*	q8h	100	>99.5	99.5	97
2	62	92.6	male	103–484	no	ineffective	*P. aeruginosa*	q6h	92	94	88	85
3	27	80	female	45–88	yes	ineffective	*E. coli*	q6h	80	91	79	54
4	46	108.2	male	81–107	no	ineffective	*E. coli*	q6h	100	97	93	84
5	70	95	female	32–36	no	ineffective	*K. pneumoniae*	q8h	90	93	83	66
6	68	65.2	female	223–648	yes	indeterminate	*E. coli*	q8h	100	100	100	100
7	63	70	female	19–23	no	ineffective	*E. coli*	q8h	58	68	47	28
8	61	78	male	73–114	no	effective	*E. coli*	q6h	100	>99.5	98.5	96
9	42	51	female	44–363	yes	ineffective	*K. pneumoniae*	q8h	100	>99.5	99.5	97.5

## Discussion

We have conducted a detailed dose-fractionation study to confirm the PK/PD index of piperacillin/tazobactam in its standard 8:1 ratio against a range of clinically relevant Gram-negative bacterial pathogens. From a case series of nine patients treated in ICU, we show that five had at least a 10% probability of not achieving the PK/PD target for 2 log_10_ kill, indicating suboptimal dosing could be a factor in treatment failure.

The best fitting index of *fT*_>MIC_ was similar as expected for β-lactam antibiotics.^14^ The targets found in our study support the ones found in other dose-fractionation studies. For bacteriostasis, targets of 42% *fT*_>MIC_,^7^ 44.9% *fT*_>MIC_^8^ and >55.1% *fT*_>MIC_^9^ were previously found, compared with 48% *fT*_>MIC_ found in this study. For 1 log_10_ kill, targets of 56% *fT*_>MIC_^7^ and 62.9% *fT*_>MIC_^8^ were found, compared with 60% *fT*_>MIC_ in this study. The added value of this study was that it was performed with six strains mostly obtained from clinical isolates, two different infusion times (30 min and 3 h) and 8:1 piperacillin:tazobactam ratio was used. The similar results to the animal study suggest that HFIM is a good replacement for PK/PD determination.^7^

Based on this small case series, piperacillin/tazobactam treatment administered by 30 min infusion in ICU patients does not seem to be effective, regardless of whether q6h or q8h is used. The DALI study showed that lower PK/PD target attainment is associated with a more negative clinical outcome.^2^ This may indicate the need to modify the current dosing schemes for ICU patients to extended or continuous infusions. Previous research indicates that prolonged or continuous infusion leads to better target attainment and clinical outcomes than intermittent infusion,^5,6,15,16^ and leads to improved outcomes on mortality.^17  18^

A limitation of this study was that it lacked adjustment for tazobactam’s influence on piperacillin susceptibility. However, we aimed to assess the standard piperacillin/tazobactam ratio for clinical extrapolation rather than determining its optimal ratio.

In our study, the piperacillin/tazobactam PK/PD model employed, designed by Lonsdale *et al.*,^3^ does not correct for haemofiltration. Subsequently, we applied a PK/PD correction parameter that was based on a PK/PD model designed for ertapenem,^12^ because of lack of more suitable data. The number of patients audited was a limited population and they had covariates that may have influenced treatment outcomes. Since MIC values for the patient isolates were unavailable in this study, the ECOFF values were used instead.^13^

More research employing larger populations should be performed to evaluate piperacillin/tazobactam effectiveness in ICU patients and determine how improved dosing schemes may achieve therapeutically effective target attainment. Ultimately, improving measures to achieve optimal dosing of piperacillin/tazobactam in the ICU is important to increase effectiveness of treatment whilst avoiding unnecessary drug exposures potentially stimulating new resistance mechanisms in bacteria. HFIMs, therefore, may be important tools to complement antimicrobial stewardship programmes in healthcare settings.

## Supplementary Material

dlae036_Supplementary_Data

## Supplementary data

Supplementary Methods and Figures S1 and S2 are available as Supplementary data at *JAC-AMR* Online.
