# Supplementary material for: Defining the pharmacokinetic/pharmacodynamic index of piperacillin/tazobactam within a hollow-fibre infection model to determine target attainment in intensive care patients
Source: JAC Antimicrob Resist. 2024 Mar 12;6(2):dlae036. doi: 10.1093/jacamr/dlae036 (PMC10928666; doi:10.1093/jacamr/dlae036)
Supplement: dlae036_Supplementary_Data [file dlae036_supplementary_data.docx]

**Supplementary**

Materials for dose fractionation assay

Antimicrobial agents were prepared from neat reagents including piperacillin white crystal powder (lot: LRAB7665; TOKU-E, USA), piperacillin sodium salt (lot: 0000189371) and tazobactam (lot: LRAB7791) supplied by Sigma-Aldrich, UK.
Six bacterial strains were used in the dose-fractionation assays: *E. coli* (ATCC25922 and DWEC107)*, Klebsiella pneumoniae* (DWKC01 and JRKC01) and *Pseudomonas aeruginosa* (SWPC02 and SWPC04). ATCC25922 was a laboratory reference strain, and the remainder were clinical isolates. The bacteria were prepared from previously prepared glycerol stock suspension, plated onto Mueller Hinton (MH) Agar plates (Sigma-Aldrich, UK) incubated under aerobic conditions at 37°C (18-24 hours) after which bacterial colonies were harvested with an inoculating loop and transferred to sterile MH Broth and further incubation in a rotating incubator (37°C) overnight. Piperacillin and tazobactam were used in an 8:1 ratio for all the experiments (MIC testing and HFIM). High, middle and low doses were determined based on predicted Cmax concentrations in a bolus scenario with128/16 mg/L for high dose, 32/8 mg/L for middle dose and 8/1 mg/L piperacillin-tazobactam for low dose.

HFIM setup

Before the start of any experiment, the cartridges were primed for 24 hours with PBS and 24 hours with Mueller Hinton Broth (M-H Broth) (supplied by Sigma-Aldrich, UK). The pumps were set to mimic a half-life of 2 hours. Halfway through the experiments, the size of the central reservoir was changed to increase the distribution of the drug through the system (run 1 to run 2). The cartridge had a volume of 28 mL, the tubing a volume of 39 mL, and the central reservoir a volume of 10 mL (run 1) or 30 mL (run 2), resulting in a total circulation volume of 77 mL (run 1) or 97 mL (run 2). The dilution rate was set at 26.6 mL/hour (run 1) or 33.6 mL/hour (run 2). All the experiments were performed at 37 ºC.

Validation pharmacokinetics experiments.

A bioassay was performed to predict the pharmacokinetic values of piperacillin-tazobactam and piperacillin alone in the HFIM. A total of four PK experiments were performed, two with the setting of run 1 and two with the setting of run 2, using 30-minute or 3-hour infusions every 6 hours. Samples were taken from the central reservoir and cartridge at 0, 5 min, 10 min, 15 min, 30 min, 1 hour, 2 hours and 4 hours after the first and fifth infusions. The piperacillin concentration of the samples was estimated using the zone of inhibition on spread Mueller Hinton Agar plates (MHA plates) with an *E. coli* laboratory strain (ATCC25922, cfu 1*10^6). A calibration curve of the logarithmic scale of varying piperacillin or piperacillin-tazobactam concentrations compared to the zone of inhibition was used. With these concentrations and nlmixr2 in R, a model with the most accurate pharmacokinetic parameters was selected (Figure S2).

**
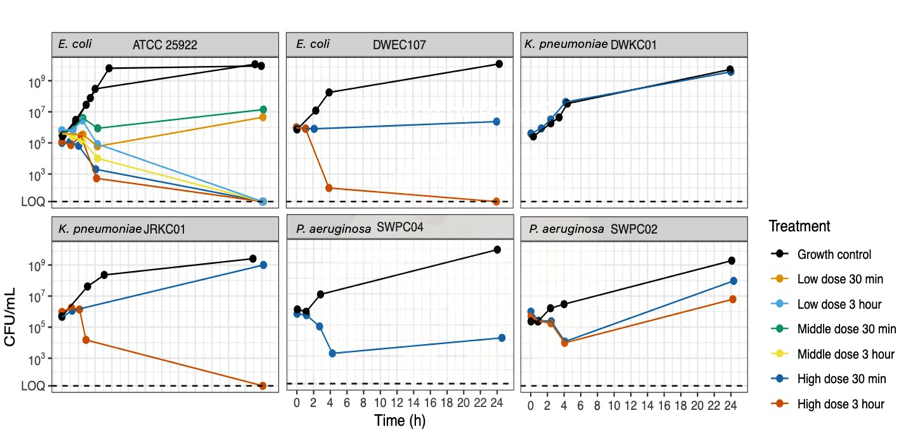
S1 Change in colony forming units (cfu)/mL over time for each hollow fibre infection model experiment.**Each graph depicts a different target organism. The colours of the lines depict different concentrations and infusion times (30 minutes or 3 hours). Low, middle, and high doses were as defined in materials and methods. Target organisms depicted - *Escherichia coli:* ATCC25922, DWEC107, *Klebsiella pneumoniae*: DWKC01, JRKC01, *Pseudomonas aeruginosa*: SWPC02, SWPC04. Drug dosing was every 6 hours and experiments were performed over 24 hours. The drug pump was initiated immediately upon inoculation. LOQ is limit of detection.


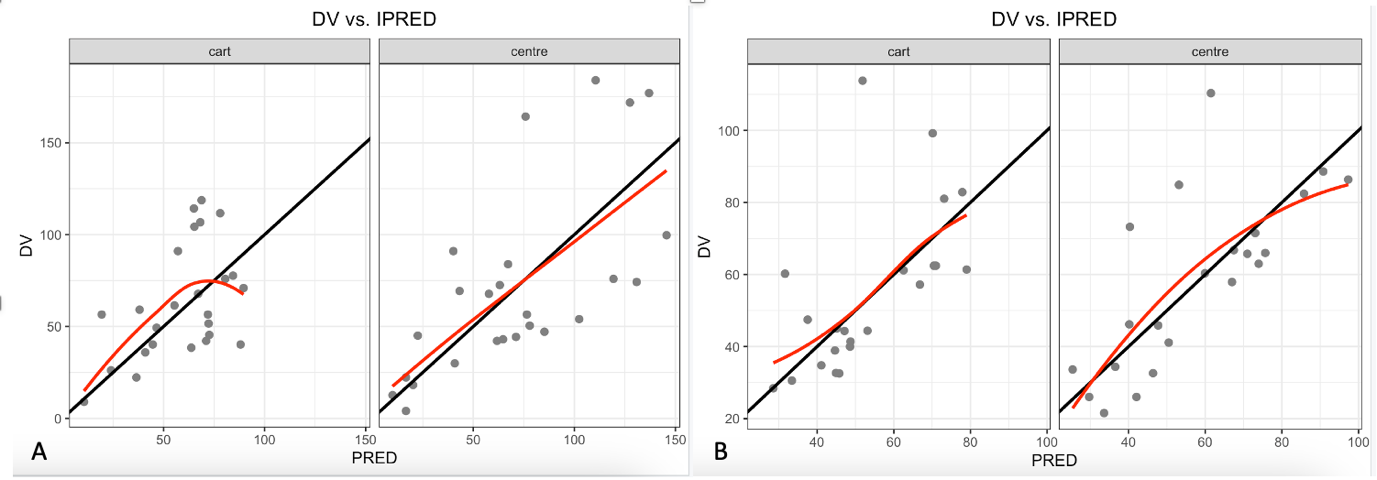


**S2. Model predicts the concentration (x-axis) using calculated PK parameters and compares it to the observed concentration (y-axis) in the HFIM.**
Black line shows the perfect fitted model, red line shows the moving average of the data. Samples were taken from the cartridge (cart) and central reservoir (centre) of the HFIM. (A) was performed with a smaller central reservoir (10 ml) and PIP- TAZ 8:1 ratio. (B) was performed with a bigger central reservoir (30 ml) and piperacillin only. DV: dependent variable. IPRED: individual prediction.
